# Clinical prediction model for bacterial co-infection in hospitalized COVID-19 patients during four waves of the pandemic

**DOI:** 10.1128/spectrum.00251-24

**Published:** 2024-09-24

**Authors:** Meital Elbaz, Itay Moshkovits, Tali Bar-On, Noam Goder, Yael Lichter, Ronen Ben-Ami

**Affiliations:** 1 Infectious Disease Unit, Tel-Aviv Sourasky Medical Center, Tel-Aviv, Israel; 2 Sackler Faculty of Medicine, Tel-Aviv University, Tel-Aviv, Israel; 3 Division of Anesthesia, Pain Management and Intensive Care, Tel-Aviv Sourasky Medical Center, Tel-Aviv, Israel; 4 Internal Medicine Department, Tel-Aviv Sourasky Medical Center, Tel-Aviv, Israel; London Health Sciences Centre, London, Ontario, Canada

**Keywords:** COVID-19, bacterial coinfection, prediction model, antibiotic stewardship

## Abstract

The reported estimates of bacterial co-infection in COVID-19 patients are highly variable. We aimed to determine the rates and risk factors of bacterial co-infection and develop a clinical prediction model to support early decision-making on antibiotic use. This is a retrospective cohort study conducted in a tertiary-level academic hospital in Israel between March 2020 and May 2022. All adult patients with severe COVID-19 who had a blood or lower respiratory specimen sent for microbiological analyses within 48 h of admission were included. The primary study endpoint was the prevalence of bacterial co-infection at the time of hospital admission. We created a prediction model using the R XGBoost package. The study cohort included 1,050 patients admitted with severe or critical COVID-19. Sixty-two patients (5.9%) had a microbiologically proven bacterial infection on admission. The variables with the greatest impact on the prediction model were age, comorbidities, functional capacity, and laboratory parameters. The model achieved perfect prediction on the training set (area under the curve = 1.0). When applied to the test dataset, the model achieved 56% and 78% specificity with the area under the receiver operating curve of 0.784. The negative and positive predictive values were 0.975 and 0.105, respectively. Applying the prediction model would have resulted in a 2.5-fold increase in appropriate antibiotic use and an 18% reduction in inappropriate use in patients with severe and critical COVID-19. The use of a clinical prediction model can support decisions to withhold empiric antimicrobial treatment at the time of hospital admission without adversely affecting patient outcomes.

**IMPORTANCE:**

Estimates of bacterial coinfection in COVID-19 patients are highly variable and depend on many factors. Patients with severe or critical COVID-19 requiring intensive care unit admission have the highest risk of infection-related complications and death. Thus, the study of the incidence and risk factors for bacterial coinfection in this population is of special interest and may help guide empiric antibiotic therapy and avoid unnecessary antimicrobial treatment. The prediction model based on clinical criteria and simple laboratory tests may be a useful tool to predict bacterial co-infection in patients hospitalized with severe COVID-19.

## INTRODUCTION

Bacterial co-infections are a well-known and potentially life-threatening complication of severe viral pneumonia, including cases caused by influenza virus (1) and, more recently, SARS-CoV-2 (2, 3). High rates of bacterial co-infection reaching 20–30% were reported for severe influenza (1, 4, 5). At the beginning of the COVID-19 pandemic, a high percentage of patients admitted to hospitals with COVID-19 were treated with empiric antimicrobial agents, a practice that was encouraged by some formal guidelines (6). However, subsequent studies showed rates of bacterial co-infection below 10% at the time of COVID-19 admission (7–9), suggesting that, in most cases, empiric antibacterial treatment is unwarranted and potentially deleterious.

Estimates of bacterial co-infection in COVID-19 are highly variable and depend on COVID-19 severity (all COVID-19 patients versus critically ill patients), patient comorbidities, diagnostic criteria used to define bacterial infection, study design, and regional epidemiology (10–12). Most reports on bacterial co-infection include all hospitalized patients with a diagnosis of COVID-19 infection (2, 7–10, 13, 14), whereas data on patients admitted to the intensive care unit (ICU) are more limited (3, 11, 15). Patients with severe or critical COVID-19 requiring ICU admission have the highest risk of infection-related complications and death (16, 17). Thus, the study of the incidence and risk factors for bacterial co-infection in this population is of special interest. Moreover, some of the studies addressing this question did not distinguish between community-acquired co-infection and hospital-acquired super-infection (2, 11). Finally, there are limited data on the rates of bacterial co-infection during COVID-19 surges caused by different SARS-CoV-2 variants, as most of the studies were done during the first wave of the pandemic (8, 18, 19).

We aimed to determine the rates and risk factors of bacterial co-infection in patients with severe and critical COVID-19 and develop a clinical prediction model to support decisions on early antibiotic use. Here, we describe the rates of bacterial co-infection at the time of admission in severe and critical COVID-19 patients admitted to a single medical center in Israel during the four time periods of the pandemic. Using this dataset, we developed a machine learning-based approach to predict bacterial co-infection among severe and critical COVID-19 patients upon hospital admission.

## MATERIALS AND METHODS

### Study design and population

This is a retrospective cohort study conducted at the Tel Aviv Sourasky Medical Center (TASMC), a tertiary-level academic hospital in Tel Aviv, Israel. The study population included adult patients admitted with severe or critical COVID-19 infection (see “definitions”), defined as positive PCR for SARS-CoV-2 from upper or lower airway specimens, between March 2020 and May 2022. All patients who had a blood or lower respiratory specimen sent for microbiological analyses within 48 h of admission were included.

The study aimed to assess the incidence of bacterial co-infection in severe and critical COVID-19 patients at the time of hospital admission and to construct a prediction model of bacterial co-infection (see “Statistical analysis”).

We included adults (age 18 years or above) with severe or critical COVID-19 infection who were admitted to COVID-19 medical wards or the ICU within the first 48 h of hospital arrival. Only patients admitted from home or long-term care facilities were included. Patients who were hospitalized for more than 48 h before COVID-19 diagnosis or transferred from another hospital were excluded.

The primary study endpoint was the prevalence of bacterial co-infection at the time of hospital admission. Explanatory variables included periods of the COVID-19 pandemic (defined below), demographic, clinical, and laboratory variables.

### Data collection and definitions

Data were retrieved from the electronic medical record system and laboratory computerized database. Collected variables included demographic data, comorbidities (quantified using the Charlson comorbidity score (20)), functional status (assessed using the Norton score), type of respiratory support (oxygen therapy, non-invasive ventilation including bi-level positive airway pressure, continuous positive airway pressure, high-flow nasal cannula, and mechanical ventilation), need for vasopressors, and laboratory data, including white blood cell count (WBC), absolute neutrophil count, lymphocyte count, platelet count, plasma levels of C-reactive protein (CRP), ferritin, and lactic acid. Laboratory data were retrieved from two time points: admission (within 24 h of hospital arrival) and follow-up (24–48 h after arrival). When a parameter was determined at both time points, the difference between the values (defined as the delta value) was calculated.

COVID-19 severity was categorized within 48 h of hospital admission according to the National Institutes of Health criteria (21) as critical (respiratory failure, septic shock, or multiorgan dysfunction) or severe [SpO_2_ <94% on room air at sea level, a ratio of arterial partial pressure of oxygen to fraction of inspired oxygen (PaO_2_/FiO_2_) <300, or respiratory rate >30 breaths/min].

Bacterial co-infection was defined as at least one of the following: (i) blood culture growing a bacterial pathogen according to the National Healthcare Safety Network (NHSN) criteria (22), including *Enterobacteriaceae*, *Pseudomonas aeruginosa*, *Acinetobacter baumannii*, *Staphylococcus aureus*, *Enterococcus faecalis*, *Enterococcus faecium*, and *Streptococcus pneumoniae* or at least two blood cultures growing beta-hemolytic streptococci; (ii) a positive culture from a lower respiratory tract specimen growing a respiratory bacterial pathogen; and (iii) a positive culture taken from pleural fluid specimen. All cases were reviewed by two study investigators (I.M. and M.E.), and their classification was agreed upon based on microbiological and clinical data.

Patients were defined as having no bacterial co-infection when blood and sputum cultures were negative during the first 72 h of admission or when blood cultures were positive only for bacteria most likely reflecting contamination (see “Microbiological methods”).

We assessed the incidence of bacterial co-infection during four time periods of the pandemic in Israel: (i) March 2020–November 2020 (Wuhan strain surge), (ii) December 2020–May 2021 (corresponding with the alpha variant wave), (iii) June 2021–November 2021 (delta variant wave), and (iv) December 2021–May 2022 (omicron variant surge).

### Microbiological methods

Blood and lower-respiratory tract cultures were performed in accordance with the American Society of Microbiology guidelines (23). Blood cultures were analyzed using the VIRTUO System (bioMerieux, Marcy l’Etoile, France). Blood contaminants were defined according to the NHSN criteria (22) as single isolation of the following bacteria: coagulase-negative staphylococci, *Micrococcus* spp., *Propionibacterium* spp.*,* viridans-type streptococci, *Bacillus* spp.*, and Corynebacterium* spp. Identification was done using the VITEK-MS MALDI-ToF System (bioMerieux, Marcy l’Etoile, France). Antimicrobial susceptibility testing was done using the VITEK-2 System (bioMerieux) and interpreted according to the Clinical and Laboratory Standards Institute criteria (24).

Semiquantitative cultures were used for invasive and non-invasive lower respiratory tract sampling, as recommended by the Infectious Diseases Society of America guidelines (25).

### Statistical analysis

Patient and disease variables were described within each patient cohort (patients with co-infection and patients without) using number (percentage) for categorical variables and median [interquartile range (IQR)] for continuous variables. Between-group differences were assessed using Fisher’s exact test for categorical variables and Student’s *t*-test and the Wilcoxon rank-sum test for normally and non-normally distributed continuous variables, respectively.

To train and validate the predictive model, the dataset was randomly partitioned into training and testing sets, each containing 50% of cases. Co-infection was set as a binary outcome variable. Explanatory variables included patient demographic variables (age, sex, body mass index), functional capacity (Norton score), comorbidities, laboratory values on admission and up to 48 h later, and interventions for respiratory and hemodynamic support. A binary classification XGBoost model was trained using the R XGBoost package. Ten-fold cross-validation on the training set was performed to optimize the variable selection and hyperparameter tuning. The number of iterations was optimized by evaluating the log loss error metric at each round, so that errors were minimized while avoiding overfitting. The area under the receiver operating curve (AUROC) was used to compare model performances and select a prediction breakpoint with balanced sensitivity and specificity values.

## RESULTS

### Study population characteristics

The study cohort included 1,050 patients admitted with severe (*n* = 742, 71%) or critical (*n* = 308, 29%) COVID-19 between March 2020 and May 2022 and (26) for whom blood cultures and/or lower respiratory tract specimens were taken during the first 72 h of admission. The median age of hospitalized patients was 75 years (IQR, 64–85 years), and 622 (59%) were male (Table 1). The median Norton score was 17 (IQR 10–20), and the median Charlson comorbidity index was 5 (IQR 3–6). Nine hundred seventy-three patients (92.7%) were admitted to medicine wards, and 77 (7.3%) were admitted to the ICU. One hundred forty-nine patients (14%) were mechanically ventilated; 138 (13%) received non-invasive ventilation; and 763 (73%) received oxygen therapy using a low-flow nasal cannula or facial mask. One hundred eight patients (10%) received treatment with vasopressors (Table 1).

**TABLE 1:** Characteristics of patients with severe and critical COVID-19^*a*^,^*b*^

	All patients (*N* = 1050)	Severe non-critical COVID-19 patients (*N* = 742)	Critical COVID1-19 patients (*N* = 308)
Age (years), median (IQR)	75 (64–85)	77 (66–86)	71 (58–71)
Male sex	622 (59%)	417 (56.2%)	205 (66.5%)
Body mass index, median (IQR)	27 (23.9–30.7)	26.8 (24–30.4)	27 (23–31)
Norton score, median (IQR)	17 (10–20)	18 (12–20)	15 (8–19)
Charlson comorbidity index, median (IQR)	5 (3–6)	5 (3–6)	4 (2–6)
Chronic lung disease	162 (16%)	122 (16.4%)	40 (13%)
Congestive heart failure	113 (11%)	72 (9.7%)	41 (13.3%)
Diabetes mellitus	357 (34%)	252 (33.9%)	105 (34%)
Chronic kidney disease	174 (17%)	115 (15.5%)	59 (12.7%)
Chronic dialysis	40 (5.6%)	11 (1.5%)	29 (9.4%)
Cancer	252 (22%)	187 (25%)	65 (21.1%)
Ischemic heart disease	117 (11%)	76 (10.2%)	41 (13.3%)
Hypertension	558 (54%)	407 (54.9%)	151 (49%)
COVID-19 severity			
Severe	742 (71%)		
Critical	308 (29%)		
Admission department			
Intensive care unit	77 (7.3%)	11 (1.5%)	66 (21.4%)
Medical wards	973 (93%)	731 (98.5%)	242 (78.6%)
Ventilation type			
Oxygen therapy	763 (73%)	742 (100%)	21 (6.8%)
Non-invasive ventilation	138 (13%)	0 (0%)	138 (44.8%)
Mechanical ventilation	149 (14%)	0 (0%)	149 (48.4%)
Vasopressors support	108 (10.3%)	0 (0%)	108 (35%)
Length of hospitalization (days)	6 (3–12)	5 (2.8–8.8)	10 (5–25)
In-hospital mortality	408 (38.9%)	211 (28.4%)	197 (63.9%)
No. of hospitalizations per period			
First wave	220 (21%)	136 (18.4%)	84 (27%)
Alpha wave	293 (28%)	190 (25.6%)	103 (33%)
Delta wave	218 (21%)	164 (22.1%)	54 (18%)
Omicron wave	319 (30%)	252 (33.9%)	67 (22%)
Laboratory data, median (IQR)			
WBC count at admission (10^3^/µl)^*c*^	7.9 (5.7–11.5)	7.6 (5.5–10.5)	9.5 (6.4–13.7)
Lymphocyte count at admission (10^3^/µl)^*c*^	0.8 (0.5–1.2)	0.8 (0.5–1.2)	0.7 (0.48–1.2)
ANC at admission^*c*^ (10^3^/µl)	6.3 (4.2–9.3)	5.9 (4.1–8.5)	7.7 (5.2–11.6)
Platelet count at admission^*c*^ (10^3^/µl)	190 (144–256)	188 (143–249)	196 (148–270)
CRP at admission^*c*^, mg/l	116 (55–170)	105 (46–155)	139 (76–197)
Ferritin at admission^*c*^, ng/ml	633 (298–1268)	576 (276–1174)	837 (404–1475)
Lactate at admission^*c*^, mmol/l	1.87 (1.43–2.51)	1.8 (1.4–2.3)	2 (1.5–3.1)
WBC count (second)^*d*^, (10^3^/µl)	8.4 (5.7–12.3)	7.7 (5.3–1.1)	10.2 (7.1–14.5)
Lymphocyte count (second)^*d*^, (10^3^/µl)	0.7 (0.5–1.1)	0.7 (0.5–1.1)	0.7 (0.4–1.1)
ANC (second)^*d*^ (10^3^/µl)	6.9 (4.4–10.5)	6 (3.9–9.5)	8.8 (5.5–12.5)
Platelet count (second),^*d*^ (10^3^/µl)	200 (142–274)	200 (140–274)	200 (147–273)
CRP (second)^*d*^ mg/l	127 (62–170)	112 (55–160)	142 (76–190)
Ferritin (second)^*d*^ ng/ml	829 (399–1528)	741 (358–1309)	1031 (501–1563)
Lactate (second)^*d*^ mmol/l	1.71 (1.35–2.27)	1.6 (1.4–2.2)	1.74 (1.35–2.44)
Microbiology			
Positive bacterial culture (all)	62 (5.9%)	31 (4.2%)	31 (10%)
Blood culture	39 (3.7%)	23 (3.1%)	16 (5.2%)
Lower respiratory tract	22 (2.1%)	7 (0.94%)	15 (4.8)
Pleural effusion	1 (0.09%)	1 (0.13%)	0

^*a*^ Categorical variables are presented as number of patients (percent) and continuous variables are presented as median (interquartile range), unless stated otherwise.

^*b*^ ANC: absolute neutrophil count.

^*c*^ The first test was taken at hospital admission (first 24 h).

^*d*^ The second test (consecutive test) was taken up to 48 h from admission.

Two hundred twenty patients (21%) were admitted during period 1 (March 2020–November 2020, first wave), 293 patients (28%) during period 2 (December 2020–May 2021, alpha wave), 218 (21%) in period 3 (June 2021–November 2021, delta wave), and 319 (30%) in period 4 (December 2021–May 2022, omicron wave).

Blood cultures were obtained from 990 patients (94.3%), and lower respiratory cultures were obtained from 53 (5%) pleural space cultures from 7 (0.7%).

**Fig 1:**
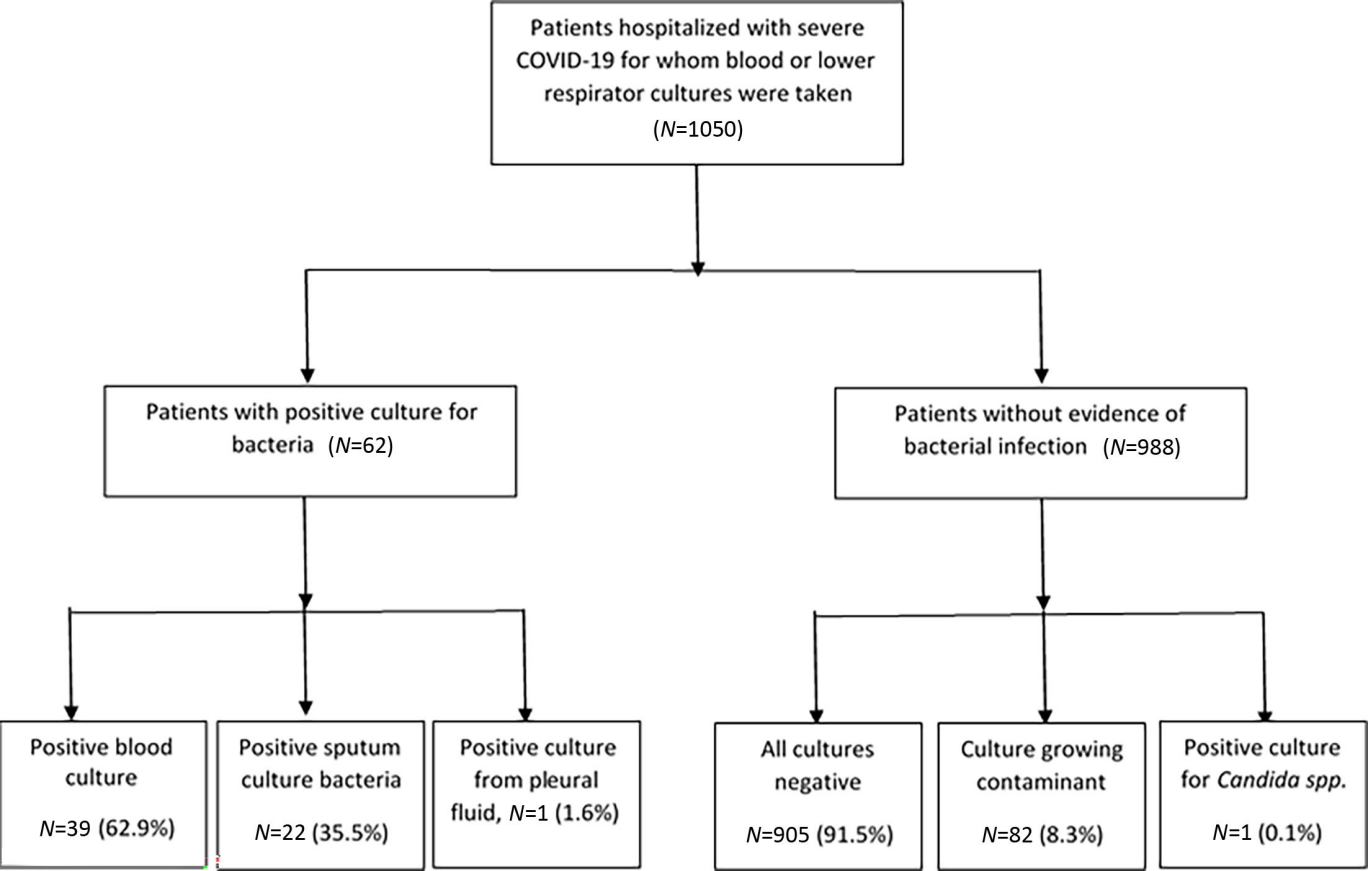
Flowchart of the positive and negative bacterial cultures obtained from the blood and respiratory tract of hospitalized severe COVID-19 patients.

Overall, 62 (5.9%) patients had a microbiologically proven bacterial infection (Table 2); 39 (62.9%) had a positive blood culture; 22 (35.5%) had a positive sputum culture; and 1 patient (1.6%) had pleural empyema growing *Streptococcus mitis* (Fig. 1).

**TABLE 2:** Bacterial isolates among 62 patients with bacterial co-infection

Bacterial isolate	Number (%)	Sample source (*N*)		
		Blood culture	Sputum culture	Pleural effusion
*Enterobacterales*	17 (27.4%)	8	9	
*Staphylococcus aureus*	12 (19.4%)	8	4	
*Streptococcus pneumoniae*	8 (12.9%)	7	1	
*Beta hemolytic streptococcus*	2 (3.2%)	2		
*Streptococcus viridans*	1 (1.6%)			1
*Enterococcus* spp.	7 (11.3%)	7		
*Pseudomonas aeruginosa*	6 (9.7%)	2	4	
*Acinetobacter baumannii*	3 (4.8%)	2	1	
*Haemophilus influenzae*	3 (4.8%)	1	2	
*Moraxella catarrhalis*	1 (1.6%)		1	
*Listeria monocytogenes*	1 (1.6%)	1		
Polymicrobial infection	1 (1.6%)	1		

The rate of bacterial co-infection was 5.0%, 5.1%, 7.7%, and 5.9% in periods 1 through 4, respectively (*P* = 0.57). The median duration of hospitalization was 6 days (IQR 3–12). In-hospital mortality was 38.8% (408/1,050).

### Bacterial co-infection prediction model

**Fig 2:**
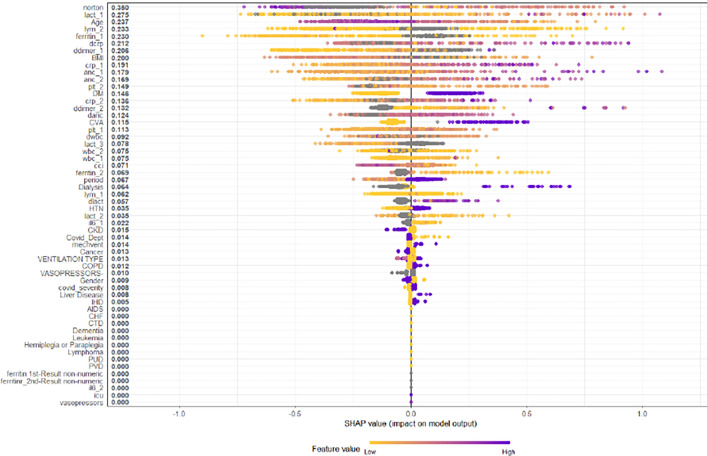
Bacterial co-infection prediction model—SHapley Additive exPlanations (SHAP) plot.

**Fig 3:**
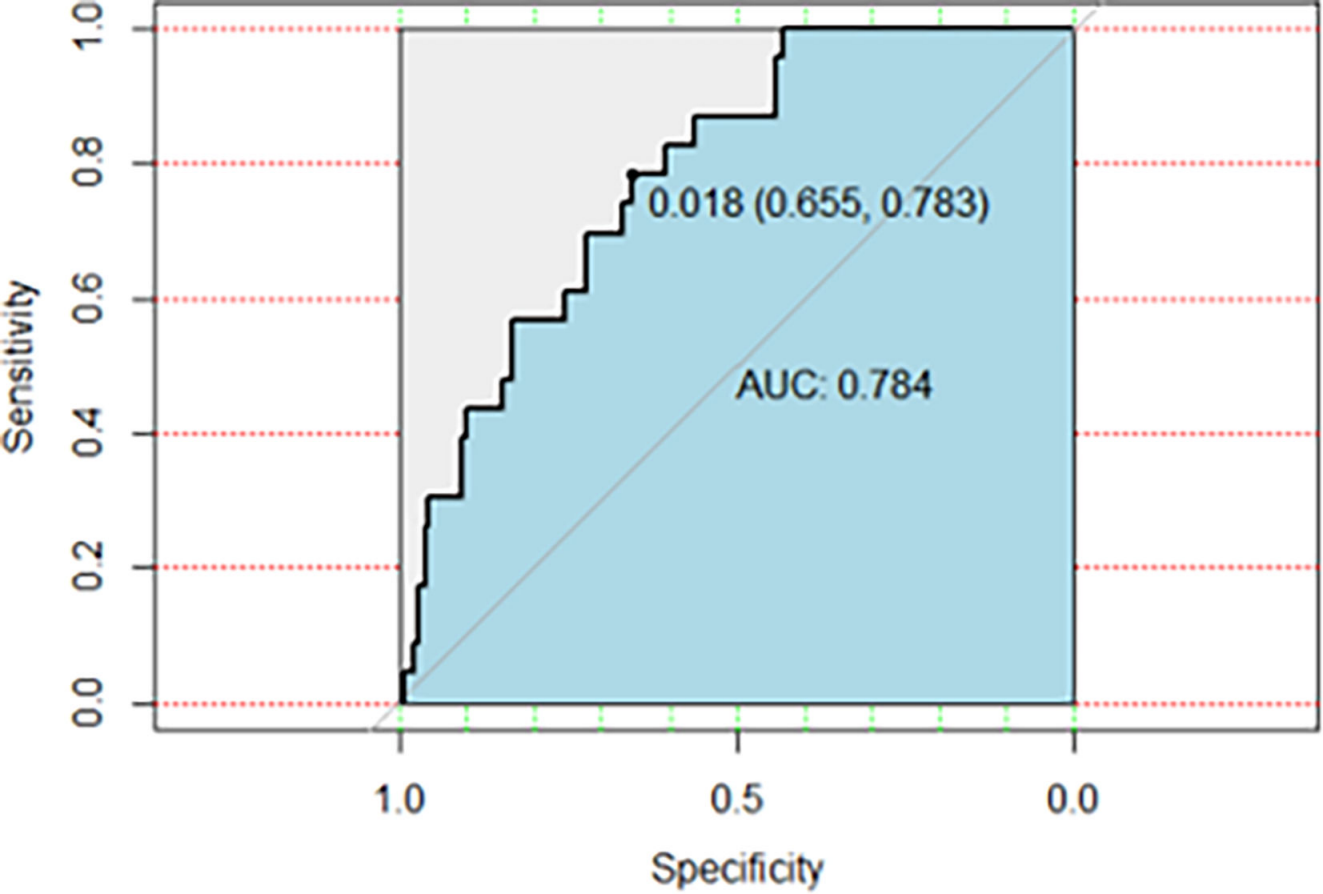
Sensitivity, specificity, and AUROC of the prediction model for bacterial co-infection.

A multivariate prediction model of bacterial co-infection was constructed with a machine learning approach using XGBoost. The database was divided randomly into training and validation datasets. The Shapley Additive exPlanations plot is shown in Fig. 2. The variables with the greatest impact on the model were patient age, comorbidities (diabetes mellitus and cerebrovascular disease), functional capacity, and laboratory parameters, notably serum lactate, ferritin, CRP, D-dimer, neutrophil, lymphocyte, and platelet counts. The model achieved perfect prediction on the training set (AUROC = 1.0). The model was then applied to the test dataset, showing a sensitivity of 56%, a specificity of 78%, and the AUROC of 0.784 (Fig. 3). The negative and positive predictive values were 0.975 and 0.105, respectively.

Of the 522 patients in the test cohort, 140 (26.8%) received antibiotic treatment, including 27.2% of patients with confirmed bacterial infections (6/22) and 26.8% of those with no infection (134/500). Simulating the prediction model on the entire dataset showed that if empiric antibiotic treatment had been guided by the model, antibiotics would have been given to 123 patients (23%): 54.5% of those with bacterial infection (15/22) and 22% of those with no infection (110/500). Thus, applying the prediction model would have resulted in a 2.5-fold increase in appropriate antibiotic use and an 18% reduction in inappropriate use in patients with severe and critical COVID-19.

## DISCUSSION

The COVID-19 pandemic was accompanied by high rates of hospital admissions due to respiratory failure. Discrimination between viral pneumonia and bacterial co-infection is challenging as the clinical and radiologic features of the two often overlap, and reliable guidance on patient selection for empiric antimicrobial treatment is lacking.

In this study, we describe the rates of community-acquired bacterial co-infection in severe and critical COVID-19 patients admitted between December 2020 and May 2022 to a single medical center in Israel. The overall rate of bacterial co-infection was 5.9% based on positive blood or lower respiratory tract cultures, and this rate did not vary significantly across periods of the pandemic associated with different SARS-CoV-2 variants.

The rate of bacterial co-infection in this study is in line with other reports (8, 27). However, we narrowed our analysis to include only severe and critical COVID-19 patients, in whom decisions regarding empiric antibiotic treatment might have the greatest impact on patient outcomes. The rate of bacterial co-infection in all COVID-19 admitted patients, including mild and moderate COVID-19 infection, is lower (2). We used a strict definition of microbiologically documented infections, whereas some previous reports defined bacterial infection based on clinical data or electronic records (7, 8, 10, 14). It is likely that some patients with bacterial infection had negative blood or sputum cultures and thus failed to meet our criteria for microbiologically documented infection. In addition, we limited our cohort to bacterial co-infection diagnosed during the 48 h period from the first presentation to the hospital and excluded patients who were transferred from other medical centers. By this, we focused on bacterial co-infection acquired in the community rather than hospital-acquired infection. Thus, 5.9% of community-acquired, microbiological-proven, bacterial co-infection might be an underestimation of the true co-infection rate, which might justify empiric antibiotic administration. Nevertheless, the fact that we did not detect a significant difference in mortality between the patients with bacterial co-infection and those without bacterial co-infection does not support broadly administering empiric antibiotics to patients with severe and critical COVID-19.

One might speculate that the rate of bacterial co-infection would be significantly different between the different periods (infection waves) of the COVID-19 pandemic. For example, the shift from hospital-based to community-based care for COVID-19, specifically during the omicron surge, resulted in a lower rate of hospital admissions (26), which might be translated into hospital presentation at later stages of the disease course during this period. In addition, some studies have reported a shorter period between symptom onset and hospital admissions in the second infection wave of COVID-19 in comparison to the first wave (28, 29). Nevertheless, we found no significant variation in the bacterial co-infection rate across periods.

When assessing factors associated with bacterial co-infection, we found that clinical variables, such as critical COVID-19 infection, ICU admission, mechanical ventilation, and vasopressor use, were correlated with bacterial infection in univariate analysis. High inflammatory markers, such as the neutrophil count and CRP levels, as well as the increase in CRP within 48 h after admission, were also correlated with bacterial infection. Similar findings were recently reported by Patton et al. (2).

Surveillance of antibiotic use data in multiple countries has shown widespread overuse of antibiotics for patients with COVID-19, with studies showing up to 80% of hospitalized COVID-19 patients receiving antibiotics (29–31). We used a machine learning approach to predict bacterial co-infection upon admission. The final model had low sensitivity and good specificity, leading to a high negative predictive value and a low positive predictive value. Use of the prediction model would have resulted in a 2.5-fold increase in appropriate antibiotic use while reducing inappropriate use by 18%. We, therefore, propose that this model can support decisions to withhold empiric antimicrobial treatment at the time of hospital admission without adversely affecting patient outcomes.

Our study has some limitations. First, this is a single-center study, which might not represent the entire population of severe/critical COVID-19 admissions. Second, although we included 1,050 patients in our cohort, we identified only 62 cases of proven bacterial co-infections, a relatively low number that might limit the statistical power of our analysis. Third, we defined co-infection as the hospital admission timing and not concerning the COVID-19 microbiological diagnosis or timing of symptoms since we do not have documentation of these data. As such, some bacterial infections that were diagnosed upon hospital admission might represent bacterial super-infections that were acquired before. Nevertheless, the definition of co-infection concerning hospital admission is common in other studies of bacterial co-infections of COVID-19 patients (2, 27).

In conclusion, rates of bacterial co-infection in severe and critical COVID-19 patients admitted to the hospital during four waves of the pandemic were relatively low. The use of our clinical prediction model can support decisions to withhold empiric antimicrobial treatment at the time of hospital admission, without adversely affecting patient outcomes.
